# Comparison of sugammadex and pyridostigmine bromide for reversal of rocuronium-induced neuromuscular blockade in short-term pediatric surgery: A prospective randomized study: Retraction

**DOI:** 10.1097/MD.0000000000020879

**Published:** 2020-06-12

The article, “Comparison of sugammadex and pyridostigmine bromide for reversal of rocuronium-induced neuromuscular blockade in short-term pediatric surgery: A prospective randomized study”,^[1]^ is being retracted. The authors reported after publication that the data used in the article, aside from secondary outcome regarding postoperative agitation in children, have already been reported in “Comparison of sugammadex and pyridostigmine bromide for reversal of rocuronium-induced neuromuscular blockade in short-term pediatric surgery: a prospective randomized study”.^[2]^
